# Supplementary material for: Cortical Mechanisms of Visual Hypersensitivity in Women at Risk for Chronic Pelvic Pain
Source: medRxiv. 2021 Jan 18:2020.12.03.20242032. Preprint. [Version 2] doi: 10.1101/2020.12.03.20242032 (PMC7836135; doi:10.1101/2020.12.03.20242032)
Supplement: Supplement 1 [file media-1.pdf]

Supplementary Material

This document contains the complete supplementary material, including both figures and tables, as referenced in the paper:

Kmiecik, M. J., Tu, F. F., Silton, R. L., Dillane, K. E., Roth, G. E., Harte, S. E., & Hellman, K. M. (2020). Cortical Mechanisms of Visual Hypersensitivity in Women at Risk for Chronic Pelvic Pain. (*full citation appears here*).

Figures and tables begin on the next page, with each display item on its own page.

Supplementary Table 1

Analysis 2 Regression Coefficient Explanations and Result Summary

Parameter				
Level One	Level Two	Explanation	Electrode	Result
Intercept b_{0i}	Intercept γ_{00}	Grand average unpleasantness ratings	All	*M* = 8, p<.001
	Menstrual Pain γ_{01}	Association between menstrual pain and unpleasantness	All	*ns*
	Somatic Symptoms γ_{02}	Association between somatic symptoms and unpleasantness	All	*ns*
	Bladder Pain γ_{03}	*Association between bladder pain and unpleasantness	All	η_p^2=.06, p=.003
Brightness Slope b_{1i}	Intercept γ_{10}	*Association between brightness intensity and unpleasantness	Oz	η_p^2=.17, p<.001
	Menstrual Pain γ_{11}	Impact of prior menstrual pain on the association between brightness intensity and unpleasantness	Oz	η_p^2=.03, p=.03
	Somatic Symptoms γ_{12}	Impact of prior somatic symptoms on the association between brightness intensity and unpleasantness	Oz	*ns*
			CP6	η_p^2=.09, p_{fdr}=.01
	Bladder Pain γ_{13}	Impact of prior experimental bladder pain on the association between unpleasantness and brightness intensity	Oz	*ns*
PSD Slope b_{2i}	Intercept γ_{20}	*How cortical excitability impacts subsequent report of visual unpleasantness	Oz	η_p^2=.07, p=.002
	Menstrual Pain γ_{21}	How prior menstrual pain impacts the association between cortical excitability and unpleasantness	Oz	*ns*
	Somatic Symptoms γ_{22}	How prior somatic symptoms impact the association between cortical excitability and unpleasantness	Oz	*ns*
			CP1	η_p^2=.06, p_{fdr}=.04
			CP6	η_p^2=.08, p_{fdr}=.02
	Bladder Pain γ_{23}	*How bladder pain affects the association between cortical excitability and unpleasantness	Oz	η_p^2=.03, p=.03

Note. Unpleasantness refers to ratings of visual unpleasantness during the visual task. ns = not significant; fdr = false discovery rate; * denotes contrasts pertinent to study's primary hypotheses.

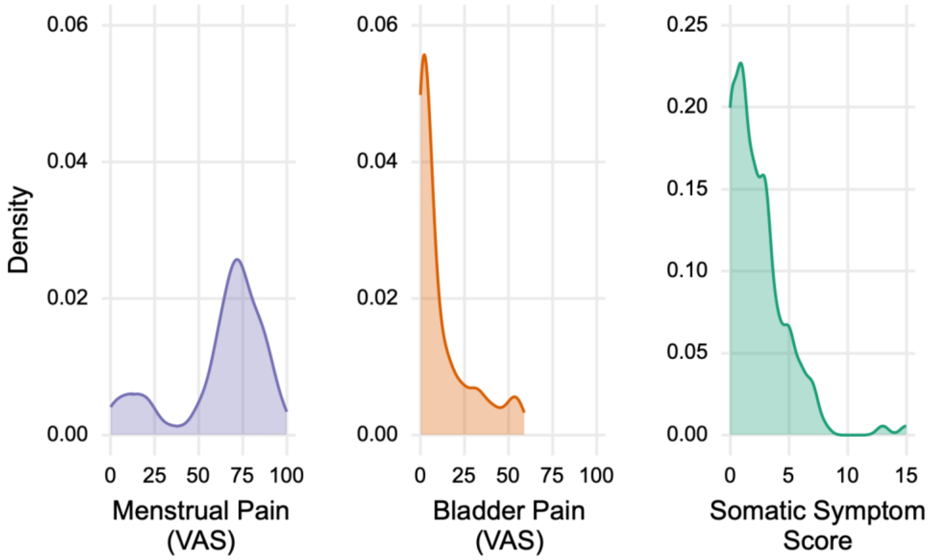

Supplementary Figure 1. **Density plots of moderating pain and somatic symptom variables in second level regression models.** The participants had a wide range of menstrual pain, bladder pain, and somatic symptom scores amenable for regression modeling. Y-axis (density) represents the concentration of points about a continuous x-axis measure. VAS = visual analog scale (0 – no pain, 100 – worst pain imaginable).

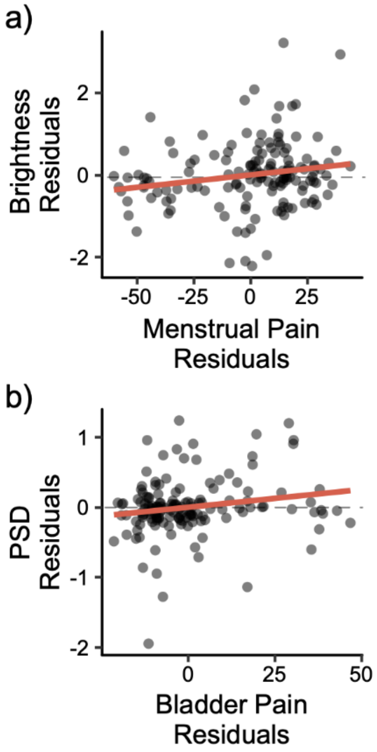

Supplementary Figure 2. **Partial regression (added variable) scatter plots depicting the moderating relationship between (a) menstrual pain and brightness slopes [b_1] and (b) bladder pain and PSD slopes [b_2] at electrode Oz.** These plots depict the unique relationship between the moderating variable (x-axis) and the dependent variable (y-axis) after accounting for the other predictors in the second level models. PSD = power spectral density (25Hz).

Supplementary Table 2

Analysis 1 Electrode-wise Multilevel Modeling Results: Predicting Power Spectral Density as a Function of Increasing Brightness

			95% CI						
Electrode	Source	*b*	*LL*	*UL*	*SE*	*SS*	*MSE*	*F*	η_p^2
C3	Intercept	-33.05	-33.74	-32.35	0.35	160549.52	18.14	8849.72	0.98
C3	Brightness	0.30	0.21	0.40	0.05	13.56	0.35	38.94	0.21
C4	Intercept	-32.93	-33.64	-32.21	0.36	159368.39	19.08	8350.83	0.98
C4	Brightness	0.40	0.29	0.51	0.05	23.71	0.43	55.05	0.27
CP1	Intercept	-31.87	-32.39	-31.35	0.26	149306.67	10.31	14478.54	0.99
CP1	Brightness	0.84	0.73	0.95	0.06	103.96	0.48	217.22	0.60
CP2	Intercept	-32.02	-32.53	-31.51	0.26	150694.86	9.81	15358.13	0.99
CP2	Brightness	0.79	0.68	0.89	0.05	90.88	0.42	215.55	0.60
CP5	Intercept	-32.39	-33.03	-31.74	0.32	154180.99	15.52	9934.66	0.99
CP5	Brightness	0.82	0.68	0.95	0.07	98.07	0.67	145.77	0.50
CP6	Intercept	-31.35	-32.10	-30.59	0.38	144443.06	21.31	6777.57	0.98
CP6	Brightness	1.13	0.99	1.27	0.07	187.01	0.74	252.54	0.63
Cz	Intercept	-31.58	-32.14	-31.02	0.28	146617.93	11.77	12453.33	0.99
Cz	Brightness	0.59	0.47	0.70	0.06	50.42	0.47	107.91	0.42
F3	Intercept	-33.44	-34.10	-32.78	0.33	164362.99	16.48	9971.85	0.99
F3	Brightness	0.28	0.19	0.37	0.04	11.52	0.27	42.04	0.22
F4	Intercept	-33.55	-34.20	-32.90	0.33	165465.80	16.06	10305.60	0.99
F4	Brightness	0.37	0.28	0.47	0.05	20.66	0.31	67.19	0.32
F7	Intercept	-33.60	-34.21	-32.99	0.31	165957.25	13.81	12021.46	0.99
F7	Brightness	0.46	0.36	0.56	0.05	30.84	0.37	82.58	0.36
F8	Intercept	-33.94	-34.52	-33.36	0.29	169314.79	12.64	13394.94	0.99
F8	Brightness	0.45	0.36	0.54	0.05	30.15	0.30	98.95	0.40

FC1	Intercept	-33.15	-33.64	-32.67	0.25	161586.36	8.96	18026.62	0.99
FC1	Brightness	0.30	0.21	0.38	0.04	13.00	0.27	48.78	0.25
FC2	Intercept	-33.42	-33.98	-32.87	0.28	164216.73	11.70	14037.12	0.99
FC2	Brightness	0.38	0.30	0.47	0.04	21.32	0.27	77.72	0.35
FC5	Intercept	-36.39	-37.06	-35.72	0.34	194656.75	16.69	11666.25	0.99
FC5	Brightness	0.43	0.33	0.52	0.05	26.58	0.31	86.56	0.37
FC6	Intercept	-35.96	-36.56	-35.36	0.30	190124.92	13.55	14028.22	0.99
FC6	Brightness	0.57	0.47	0.67	0.05	47.46	0.39	120.74	0.45
Fp1	Intercept	-33.59	-34.28	-32.91	0.34	165899.40	17.46	9500.04	0.98
Fp1	Brightness	0.71	0.59	0.83	0.06	73.90	0.56	132.13	0.48
Fp2	Intercept	-33.85	-34.51	-33.19	0.33	168419.31	16.37	10290.52	0.99
Fp2	Brightness	0.69	0.56	0.83	0.07	70.26	0.68	103.83	0.42
FT10	Intercept	-34.05	-34.57	-33.53	0.26	170412.26	10.19	16727.84	0.99
FT10	Brightness	0.61	0.49	0.73	0.06	54.89	0.51	108.27	0.43
FT9	Intercept	-33.19	-33.69	-32.69	0.26	161930.75	9.58	16900.83	0.99
FT9	Brightness	0.50	0.40	0.60	0.05	36.83	0.39	94.05	0.39
Fz	Intercept	-32.53	-32.99	-32.06	0.23	155525.37	8.06	19300.29	0.99
Fz	Brightness	0.29	0.21	0.37	0.04	12.23	0.24	50.19	0.26
O1	Intercept	-26.04	-26.83	-25.25	0.40	99674.02	23.37	4264.77	0.97
O1	Brightness	1.51	1.35	1.66	0.08	333.19	0.86	386.08	0.73
O2	Intercept	-25.24	-26.19	-24.28	0.48	93641.62	34.36	2725.20	0.95
O2	Brightness	1.74	1.57	1.91	0.09	446.71	1.09	410.68	0.74
Oz	Intercept	-23.76	-24.68	-22.84	0.47	82992.47	31.92	2600.06	0.95
Oz	Brightness	2.05	1.87	2.23	0.09	617.64	1.22	504.68	0.78
P3	Intercept	-28.91	-29.57	-28.26	0.33	122874.54	16.20	7583.54	0.98
P3	Brightness	1.08	0.93	1.22	0.07	170.46	0.82	207.32	0.59
P4	Intercept	-27.08	-27.83	-26.33	0.38	107831.97	21.16	5096.26	0.97
P4	Brightness	1.18	1.04	1.32	0.07	203.70	0.72	283.55	0.66

P7	Intercept	-28.41	-29.16	-27.66	0.38	118621.46	21.12	5617.08	0.97
P7	Brightness	1.40	1.25	1.56	0.08	289.50	0.87	331.65	0.69
P8	Intercept	-27.23	-28.02	-26.44	0.40	109028.88	23.47	4646.25	0.97
P8	Brightness	1.35	1.20	1.50	0.08	269.01	0.84	321.58	0.69
Pz	Intercept	-27.99	-28.68	-27.31	0.35	115184.29	17.76	6484.82	0.98
Pz	Brightness	0.99	0.83	1.14	0.08	143.36	0.89	160.59	0.52
T7	Intercept	-32.69	-33.35	-32.03	0.34	157068.91	16.51	9512.60	0.98
T7	Brightness	0.59	0.48	0.70	0.06	50.65	0.45	111.63	0.43
T8	Intercept	-33.08	-33.73	-32.42	0.33	160826.65	16.17	9946.21	0.99
T8	Brightness	0.81	0.68	0.93	0.06	95.44	0.62	155.18	0.52
TP10	Intercept	-30.83	-31.54	-30.11	0.36	139682.32	19.46	7178.68	0.98
TP10	Brightness	1.26	1.11	1.42	0.08	233.98	0.91	258.47	0.64
TP9	Intercept	-31.11	-31.78	-30.43	0.34	142226.41	17.32	8213.65	0.98
TP9	Brightness	0.91	0.77	1.05	0.07	122.67	0.75	164.05	0.53

Note. Both FDR corrected and uncorrected p values were < .001; df for the numerator and denominator were 1 and 46, respectively; LL = lower level; UL = upper level.

Supplementary Table 3

Analysis 2 Electrode-wise Multilevel Modeling Results: Estimating Brain-Behavior Relationships and the Moderating Effects of Pain and Somatic Symptoms

				95% CI								
Electrode	Model	Source	*b*	*LL*	*UL*	*SE*	*SS*	*MSE*	*F*	*p*	p_{fdr}	η_p^2
All	Intercept	Intercept	7.997	7.367	8.627	0.319	9401.60	14.92	630.0	< . 001	< . 001	.815
All	Intercept	Bladder Pain	0.064	0.022	0.107	0.021	134.33	14.92	9.0	.003	.003	.059
All	Intercept	Menstrual Pain	0.012	-0.013	0.038	0.013	13.99	14.92	0.9	.335	.335	.007
All	Intercept	Somatic Symptoms	-0.135	-0.411	0.140	0.139	14.13	14.92	0.9	.332	.332	.007
C3	Brightness	Intercept	0.559	0.411	0.706	0.075	45.92	0.82	56.1	< . 001	< . 001	.282
C3	Brightness	Bladder Pain	0.000	-0.010	0.010	0.005	0.00	0.82	0.0	.985	.985	< . 001
C3	Brightness	Menstrual Pain	0.005	-0.001	0.011	0.003	2.35	0.82	2.9	.093	.153	.02
C3	Brightness	Somatic Symptoms	-0.028	-0.093	0.036	0.033	0.61	0.82	0.7	.389	.817	.005
C4	Brightness	Intercept	0.451	0.297	0.606	0.078	29.96	0.90	33.4	< . 001	< . 001	.189
C4	Brightness	Bladder Pain	-0.005	-0.015	0.005	0.005	0.82	0.90	0.9	.34	.985	.006
C4	Brightness	Menstrual Pain	0.006	0.000	0.012	0.003	3.40	0.90	3.8	.054	.132	.026
C4	Brightness	Somatic Symptoms	-0.053	-0.121	0.014	0.034	2.18	0.90	2.4	.121	.58	.017
CP1	Brightness	Intercept	0.492	0.351	0.633	0.071	35.61	0.75	47.7	< . 001	< . 001	.25
CP1	Brightness	Bladder Pain	-0.002	-0.011	0.008	0.005	0.10	0.75	0.1	.717	.985	.001
CP1	Brightness	Menstrual Pain	0.005	-0.001	0.010	0.003	1.99	0.75	2.7	.105	.153	.018
CP1	Brightness	Somatic Symptoms	0.013	-0.048	0.075	0.031	0.14	0.75	0.2	.669	.93	.001
CP2	Brightness	Intercept	0.448	0.256	0.640	0.097	29.50	1.38	21.3	< . 001	< . 001	.13
CP2	Brightness	Bladder Pain	0.001	-0.012	0.014	0.007	0.01	1.38	0.0	.926	.985	< . 001
CP2	Brightness	Menstrual Pain	0.008	0.001	0.016	0.004	6.26	1.38	4.5	.035	.126	.031
CP2	Brightness	Somatic Symptoms	-0.078	-0.162	0.006	0.042	4.71	1.38	3.4	.067	.535	.023
CP5	Brightness	Intercept	0.434	0.262	0.606	0.087	27.64	1.11	24.8	< . 001	< . 001	.148
CP5	Brightness	Bladder Pain	0.002	-0.010	0.013	0.006	0.09	1.11	0.1	.78	.985	.001
CP5	Brightness	Menstrual Pain	0.004	-0.003	0.011	0.004	1.55	1.11	1.4	.239	.278	.01

CP5	Brightness	Somatic Symptoms	-0.029	-0.104	0.046	0.038	0.64	1.11	0.6	.448	.843	.004
CP6	Brightness	Intercept	0.428	0.240	0.617	0.095	26.96	1.34	20.2	< . 001	< . 001	.124
CP6	Brightness	Bladder Pain	0.005	-0.007	0.018	0.006	0.96	1.34	0.7	.398	.985	.005
CP6	Brightness	Menstrual Pain	0.006	-0.001	0.014	0.004	3.71	1.34	2.8	.098	.153	.019
CP6	Brightness	Somatic Symptoms	-0.157	-0.239	-0.074	0.042	18.87	1.34	14.1	< . 001	.008	.09
Cz	Brightness	Intercept	0.481	0.333	0.629	0.075	33.98	0.82	41.2	< . 001	< . 001	.224
Cz	Brightness	Bladder Pain	-0.002	-0.012	0.008	0.005	0.09	0.82	0.1	.747	.985	.001
Cz	Brightness	Menstrual Pain	0.004	-0.002	0.010	0.003	1.63	0.82	2.0	.162	.215	.014
Cz	Brightness	Somatic Symptoms	-0.018	-0.083	0.046	0.033	0.25	0.82	0.3	.579	.896	.002
F3	Brightness	Intercept	0.562	0.423	0.700	0.070	46.37	0.72	64.2	< . 001	< . 001	.31
F3	Brightness	Bladder Pain	-0.001	-0.010	0.009	0.005	0.01	0.72	0.0	.913	.985	< . 001
F3	Brightness	Menstrual Pain	0.009	0.003	0.014	0.003	6.70	0.72	9.3	.003	.089	.061
F3	Brightness	Somatic Symptoms	-0.008	-0.068	0.053	0.031	0.04	0.72	0.1	.804	.934	< . 001
F4	Brightness	Intercept	0.485	0.357	0.613	0.065	34.58	0.62	56.1	< . 001	< . 001	.282
F4	Brightness	Bladder Pain	0.002	-0.006	0.011	0.004	0.16	0.62	0.3	.61	.985	.002
F4	Brightness	Menstrual Pain	0.005	0.000	0.010	0.003	2.15	0.62	3.5	.064	.134	.024
F4	Brightness	Somatic Symptoms	-0.032	-0.088	0.024	0.028	0.79	0.62	1.3	.259	.723	.009
F7	Brightness	Intercept	0.515	0.363	0.667	0.077	39.00	0.87	44.7	< . 001	< . 001	.238
F7	Brightness	Bladder Pain	0.007	-0.004	0.017	0.005	1.43	0.87	1.6	.203	.985	.011
F7	Brightness	Menstrual Pain	0.004	-0.002	0.010	0.003	1.40	0.87	1.6	.208	.266	.011
F7	Brightness	Somatic Symptoms	-0.016	-0.083	0.051	0.034	0.20	0.87	0.2	.635	.924	.002
F8	Brightness	Intercept	0.450	0.309	0.590	0.071	29.75	0.74	40.0	< . 001	< . 001	.219
F8	Brightness	Bladder Pain	0.003	-0.006	0.013	0.005	0.37	0.74	0.5	.484	.985	.003
F8	Brightness	Menstrual Pain	0.002	-0.003	0.008	0.003	0.55	0.74	0.7	.392	.433	.005
F8	Brightness	Somatic Symptoms	0.006	-0.056	0.067	0.031	0.03	0.74	0.0	.85	.934	< . 001
FC1	Brightness	Intercept	0.685	0.490	0.879	0.098	68.91	1.42	48.5	< . 001	< . 001	.253
FC1	Brightness	Bladder Pain	0.007	-0.006	0.020	0.007	1.62	1.42	1.1	.288	.985	.008
FC1	Brightness	Menstrual Pain	0.008	0.001	0.016	0.004	6.40	1.42	4.5	.036	.126	.031
FC1	Brightness	Somatic Symptoms	-0.006	-0.091	0.079	0.043	0.03	1.42	0.0	.886	.934	< . 001
FC2	Brightness	Intercept	0.526	0.386	0.666	0.071	40.72	0.74	55.2	< . 001	< . 001	.278
FC2	Brightness	Bladder Pain	0.003	-0.007	0.012	0.005	0.22	0.74	0.3	.585	.985	.002

FC2	Brightness	Menstrual Pain	0.003	-0.002	0.009	0.003	1.08	0.74	1.5	.228	.278	.01
FC2	Brightness	Somatic Symptoms	-0.010	-0.071	0.052	0.031	0.07	0.74	0.1	.756	.934	.001
FC5	Brightness	Intercept	0.479	0.349	0.608	0.065	33.69	0.63	53.6	< . 001	< . 001	.273
FC5	Brightness	Bladder Pain	0.002	-0.007	0.011	0.004	0.13	0.63	0.2	.654	.985	.001
FC5	Brightness	Menstrual Pain	0.004	-0.001	0.010	0.003	1.68	0.63	2.7	.104	.153	.018
FC5	Brightness	Somatic Symptoms	-0.018	-0.075	0.038	0.029	0.26	0.63	0.4	.519	.896	.003
FC6	Brightness	Intercept	0.527	0.394	0.660	0.067	40.86	0.67	61.4	< . 001	< . 001	.3
FC6	Brightness	Bladder Pain	0.001	-0.008	0.010	0.005	0.05	0.67	0.1	.789	.985	.001
FC6	Brightness	Menstrual Pain	0.002	-0.004	0.007	0.003	0.22	0.67	0.3	.565	.583	.002
FC6	Brightness	Somatic Symptoms	-0.016	-0.074	0.042	0.029	0.20	0.67	0.3	.588	.896	.002
Fp1	Brightness	Intercept	0.524	0.361	0.688	0.083	40.43	1.01	40.1	< . 001	< . 001	.219
Fp1	Brightness	Bladder Pain	0.001	-0.010	0.012	0.006	0.03	1.01	0.0	.863	.985	< . 001
Fp1	Brightness	Menstrual Pain	0.008	0.001	0.015	0.003	5.82	1.01	5.8	.018	.113	.039
Fp1	Brightness	Somatic Symptoms	-0.072	-0.144	-0.001	0.036	4.01	1.01	4.0	.048	.514	.027
Fp2	Brightness	Intercept	0.428	0.279	0.578	0.076	26.98	0.84	32.0	< . 001	< . 001	.183
Fp2	Brightness	Bladder Pain	0.004	-0.006	0.014	0.005	0.44	0.84	0.5	.474	.985	.004
Fp2	Brightness	Menstrual Pain	0.004	-0.002	0.010	0.003	1.76	0.84	2.1	.151	.211	.014
Fp2	Brightness	Somatic Symptoms	-0.011	-0.077	0.054	0.033	0.10	0.84	0.1	.732	.934	.001
FT10	Brightness	Intercept	0.451	0.302	0.600	0.076	29.93	0.84	35.7	< . 001	< . 001	.2
FT10	Brightness	Bladder Pain	0.005	-0.005	0.015	0.005	0.87	0.84	1.0	.31	.985	.007
FT10	Brightness	Menstrual Pain	0.005	-0.001	0.011	0.003	2.29	0.84	2.7	.1	.153	.019
FT10	Brightness	Somatic Symptoms	0.002	-0.063	0.067	0.033	0.00	0.84	0.0	.955	.955	< . 001
FT9	Brightness	Intercept	0.542	0.402	0.682	0.071	43.16	0.74	58.3	< . 001	< . 001	.289
FT9	Brightness	Bladder Pain	0.003	-0.006	0.013	0.005	0.32	0.74	0.4	.512	.985	.003
FT9	Brightness	Menstrual Pain	0.006	0.000	0.012	0.003	3.20	0.74	4.3	.039	.126	.029
FT9	Brightness	Somatic Symptoms	-0.046	-0.107	0.015	0.031	1.64	0.74	2.2	.139	.58	.015
Fz	Brightness	Intercept	0.515	0.373	0.657	0.072	39.02	0.76	51.5	< . 001	< . 001	.265
Fz	Brightness	Bladder Pain	0.005	-0.005	0.014	0.005	0.67	0.76	0.9	.35	.985	.006
Fz	Brightness	Menstrual Pain	0.006	0.000	0.011	0.003	2.90	0.76	3.8	.052	.132	.026
Fz	Brightness	Somatic Symptoms	-0.029	-0.091	0.033	0.031	0.67	0.76	0.9	.35	.817	.006
O1	Brightness	Intercept	0.459	0.301	0.618	0.080	31.03	0.95	32.7	< . 001	< . 001	.186

O1	Brightness	Bladder Pain	-0.001	-0.011	0.010	0.005	0.01	0.95	0.0	.905	.985	.
O1	Brightness	Menstrual Pain	0.008	0.002	0.015	0.003	6.42	0.95	6.8	.01	.109	.045
O1	Brightness	Somatic Symptoms	-0.059	-0.129	0.010	0.035	2.69	0.95	2.8	.094	.58	.019
O2	Brightness	Intercept	0.410	0.258	0.562	0.077	24.69	0.87	28.4	< . 001	< . 001	.166
O2	Brightness	Bladder Pain	0.005	-0.005	0.016	0.005	0.94	0.87	1.1	.3	.985	.008
O2	Brightness	Menstrual Pain	0.004	-0.002	0.010	0.003	1.19	0.87	1.4	.243	.278	.01
O2	Brightness	Somatic Symptoms	-0.031	-0.097	0.036	0.034	0.74	0.87	0.8	.359	.817	.006
Oz	Brightness	Intercept	0.357	0.225	0.489	0.067	18.75	0.66	28.6	< . 001	< . 001	.167
Oz	Brightness	Bladder Pain	-0.003	-0.012	0.006	0.004	0.27	0.66	0.4	.519	.985	.003
Oz	Brightness	Menstrual Pain	0.006	0.001	0.011	0.003	3.32	0.66	5.1	.026	.118	.034
Oz	Brightness	Somatic Symptoms	-0.032	-0.090	0.025	0.029	0.80	0.66	1.2	.271	.723	.008
P3	Brightness	Intercept	0.453	0.305	0.601	0.075	30.18	0.82	36.7	< . 001	< . 001	.204
P3	Brightness	Bladder Pain	0.000	-0.010	0.010	0.005	0.01	0.82	0.0	.933	.985	< . 001
P3	Brightness	Menstrual Pain	0.006	0.000	0.012	0.003	3.22	0.82	3.9	.05	.132	.027
P3	Brightness	Somatic Symptoms	-0.042	-0.106	0.023	0.033	1.35	0.82	1.6	.202	.718	.011
P4	Brightness	Intercept	0.481	0.345	0.616	0.069	34.01	0.69	49.2	< . 001	< . 001	.256
P4	Brightness	Bladder Pain	0.006	-0.003	0.015	0.005	1.24	0.69	1.8	.182	.985	.012
P4	Brightness	Menstrual Pain	0.006	0.001	0.012	0.003	3.71	0.69	5.4	.022	.117	.036
P4	Brightness	Somatic Symptoms	-0.044	-0.103	0.015	0.030	1.48	0.69	2.1	.145	.58	.015
P7	Brightness	Intercept	0.425	0.279	0.571	0.074	26.51	0.80	33.0	< . 001	< . 001	.188
P7	Brightness	Bladder Pain	-0.003	-0.013	0.007	0.005	0.28	0.80	0.3	.559	.985	.002
P7	Brightness	Menstrual Pain	0.008	0.002	0.014	0.003	6.34	0.80	7.9	.006	.09	.052
P7	Brightness	Somatic Symptoms	-0.027	-0.091	0.037	0.032	0.55	0.80	0.7	.408	.817	.005
P8	Brightness	Intercept	0.437	0.298	0.576	0.070	28.11	0.73	38.6	< . 001	< . 001	.213
P8	Brightness	Bladder Pain	0.003	-0.006	0.012	0.005	0.29	0.73	0.4	.525	.985	.003
P8	Brightness	Menstrual Pain	0.007	0.001	0.012	0.003	4.20	0.73	5.8	.018	.113	.039
P8	Brightness	Somatic Symptoms	-0.078	-0.139	-0.017	0.031	4.68	0.73	6.4	.012	.197	.043
Pz	Brightness	Intercept	0.425	0.291	0.560	0.068	26.56	0.68	39.1	< . 001	< . 001	.215
Pz	Brightness	Bladder Pain	0.000	-0.009	0.009	0.005	0.00	0.68	0.0	.964	.985	< . 001
Pz	Brightness	Menstrual Pain	0.005	0.000	0.010	0.003	2.32	0.68	3.4	.067	.134	.023
Pz	Brightness	Somatic Symptoms	-0.017	-0.076	0.042	0.030	0.22	0.68	0.3	.568	.896	.002

T7	Brightness	Intercept	0.341	0.147	0.535	0.098	17.09	1.41	12.1	.001	.001	.078
T7	Brightness	Bladder Pain	0.000	-0.013	0.013	0.007	0.00	1.41	0.0	.979	.985	< . 001
T7	Brightness	Menstrual Pain	0.001	-0.007	0.009	0.004	0.12	1.41	0.1	.773	.773	.001
T7	Brightness	Somatic Symptoms	-0.007	-0.091	0.078	0.043	0.03	1.41	0.0	.877	.934	< . 001
T8	Brightness	Intercept	0.459	0.312	0.605	0.074	30.94	0.81	38.4	< . 001	< . 001	.211
T8	Brightness	Bladder Pain	0.004	-0.006	0.013	0.005	0.41	0.81	0.5	.478	.985	.004
T8	Brightness	Menstrual Pain	0.006	0.000	0.011	0.003	2.75	0.81	3.4	.067	.134	.023
T8	Brightness	Somatic Symptoms	0.009	-0.055	0.073	0.032	0.06	0.81	0.1	.779	.934	.001
TP10	Brightness	Intercept	0.410	0.261	0.558	0.075	24.67	0.83	29.7	< . 001	< . 001	.172
TP10	Brightness	Bladder Pain	0.004	-0.006	0.014	0.005	0.53	0.83	0.6	.427	.985	.004
TP10	Brightness	Menstrual Pain	0.002	-0.004	0.008	0.003	0.40	0.83	0.5	.49	.523	.003
TP10	Brightness	Somatic Symptoms	0.004	-0.061	0.069	0.033	0.01	0.83	0.0	.905	.934	< . 001
TP9	Brightness	Intercept	0.617	0.375	0.860	0.123	56.05	2.22	25.3	< . 001	< . 001	.15
TP9	Brightness	Bladder Pain	0.007	-0.009	0.023	0.008	1.59	2.22	0.7	.398	.985	.005
TP9	Brightness	Menstrual Pain	0.008	-0.002	0.018	0.005	6.17	2.22	2.8	.097	.153	.019
TP9	Brightness	Somatic Symptoms	-0.061	-0.167	0.045	0.054	2.85	2.22	1.3	.258	.723	.009
C3	PSD	Intercept	0.010	-0.156	0.175	0.084	0.01	1.03	0.0	.91	.945	< . 001
C3	PSD	Bladder Pain	0.001	-0.010	0.012	0.006	0.05	1.03	0.1	.819	.974	< . 001
C3	PSD	Menstrual Pain	0.000	-0.007	0.006	0.003	0.02	1.03	0.0	.89	.968	< . 001
C3	PSD	Somatic Symptoms	-0.023	-0.096	0.049	0.037	0.42	1.03	0.4	.522	.93	.003
C4	PSD	Intercept	0.225	0.079	0.370	0.074	7.42	0.80	9.3	.003	.021	.061
C4	PSD	Bladder Pain	0.009	-0.001	0.019	0.005	2.54	0.80	3.2	.076	.796	.022
C4	PSD	Menstrual Pain	0.002	-0.004	0.008	0.003	0.34	0.80	0.4	.516	.968	.003
C4	PSD	Somatic Symptoms	-0.022	-0.086	0.041	0.032	0.38	0.80	0.5	.491	.93	.003
CP1	PSD	Intercept	0.041	-0.166	0.248	0.105	0.25	1.62	0.2	.697	.779	.001
CP1	PSD	Bladder Pain	-0.011	-0.025	0.003	0.007	3.63	1.62	2.2	.137	.796	.015
CP1	PSD	Menstrual Pain	0.003	-0.005	0.012	0.004	1.09	1.62	0.7	.412	.968	.005
CP1	PSD	Somatic Symptoms	-0.140	-0.231	-0.050	0.046	15.13	1.62	9.4	.003	.043	.061
CP2	PSD	Intercept	0.047	-0.117	0.211	0.083	0.33	1.01	0.3	.569	.701	.002
CP2	PSD	Bladder Pain	0.005	-0.006	0.016	0.006	0.72	1.01	0.7	.4	.916	.005
CP2	PSD	Menstrual Pain	-0.005	-0.012	0.001	0.003	2.73	1.01	2.7	.103	.968	.019

CP2	PSD	Somatic Symptoms	0.051	-0.021	0.122	0.036	1.97	1.01	1.9	.165	.794	.013
CP5	PSD	Intercept	0.125	-0.024	0.274	0.076	2.29	0.84	2.7	.1	.268	.019
CP5	PSD	Bladder Pain	0.001	-0.009	0.011	0.005	0.01	0.84	0.0	.909	.974	< . 001
CP5	PSD	Menstrual Pain	0.001	-0.005	0.007	0.003	0.03	0.84	0.0	.858	.968	< . 001
CP5	PSD	Somatic Symptoms	-0.035	-0.100	0.031	0.033	0.92	0.84	1.1	.297	.794	.008
CP6	PSD	Intercept	0.202	0.091	0.314	0.056	6.01	0.47	12.9	< . 001	.012	.083
CP6	PSD	Bladder Pain	-0.001	-0.009	0.006	0.004	0.05	0.47	0.1	.741	.974	.001
CP6	PSD	Menstrual Pain	0.000	-0.005	0.004	0.002	0.00	0.47	0.0	.968	.968	< . 001
CP6	PSD	Somatic Symptoms	0.088	0.040	0.137	0.025	5.99	0.47	12.8	< . 001	.015	.082
Cz	PSD	Intercept	0.062	-0.091	0.215	0.077	0.57	0.88	0.6	.423	.618	.005
Cz	PSD	Bladder Pain	0.006	-0.004	0.017	0.005	1.31	0.88	1.5	.225	.796	.01
Cz	PSD	Menstrual Pain	0.000	-0.006	0.006	0.003	0.00	0.88	0.0	.948	.968	< . 001
Cz	PSD	Somatic Symptoms	0.009	-0.058	0.076	0.034	0.06	0.88	0.1	.79	.931	< . 001
F3	PSD	Intercept	0.039	-0.146	0.224	0.094	0.22	1.29	0.2	.68	.779	.001
F3	PSD	Bladder Pain	0.001	-0.011	0.014	0.006	0.05	1.29	0.0	.849	.974	< . 001
F3	PSD	Menstrual Pain	0.001	-0.007	0.008	0.004	0.03	1.29	0.0	.88	.968	< . 001
F3	PSD	Somatic Symptoms	-0.053	-0.134	0.028	0.041	2.15	1.29	1.7	.199	.794	.012
F4	PSD	Intercept	0.176	-0.024	0.376	0.101	4.57	1.50	3.0	.083	.242	.021
F4	PSD	Bladder Pain	-0.001	-0.015	0.012	0.007	0.05	1.50	0.0	.856	.974	< . 001
F4	PSD	Menstrual Pain	-0.002	-0.010	0.006	0.004	0.43	1.50	0.3	.594	.968	.002
F4	PSD	Somatic Symptoms	-0.017	-0.104	0.070	0.044	0.22	1.50	0.1	.7	.931	.001
F7	PSD	Intercept	0.150	-0.044	0.343	0.098	3.29	1.41	2.3	.129	.283	.016
F7	PSD	Bladder Pain	0.003	-0.010	0.016	0.007	0.22	1.41	0.2	.692	.974	.001
F7	PSD	Menstrual Pain	-0.001	-0.008	0.007	0.004	0.03	1.41	0.0	.893	.968	< . 001
F7	PSD	Somatic Symptoms	-0.003	-0.088	0.081	0.043	0.01	1.41	0.0	.941	.941	< . 001
F8	PSD	Intercept	0.195	0.019	0.371	0.089	5.61	1.17	4.8	.03	.16	.033
F8	PSD	Bladder Pain	0.002	-0.010	0.014	0.006	0.10	1.17	0.1	.767	.974	.001
F8	PSD	Menstrual Pain	0.002	-0.005	0.009	0.004	0.25	1.17	0.2	.642	.968	.002
F8	PSD	Somatic Symptoms	-0.042	-0.119	0.035	0.039	1.34	1.17	1.2	.285	.794	.008
FC1	PSD	Intercept	-0.210	-0.594	0.173	0.194	6.50	5.54	1.2	.28	.472	.008
FC1	PSD	Bladder Pain	-0.017	-0.043	0.008	0.013	9.89	5.54	1.8	.184	.796	.012

FC1	PSD	Menstrual Pain	-0.007	-0.022	0.009	0.008	4.08	5.54	0.7	.392	.968	.005
FC1	PSD	Somatic Symptoms	0.042	-0.126	0.210	0.085	1.36	5.54	0.2	.62	.931	.002
FC2	PSD	Intercept	0.119	-0.070	0.308	0.096	2.09	1.35	1.6	.215	.404	.011
FC2	PSD	Bladder Pain	-0.007	-0.020	0.005	0.006	1.80	1.35	1.3	.249	.796	.009
FC2	PSD	Menstrual Pain	0.004	-0.003	0.012	0.004	1.66	1.35	1.2	.268	.968	.009
FC2	PSD	Somatic Symptoms	-0.035	-0.117	0.048	0.042	0.93	1.35	0.7	.407	.868	.005
FC5	PSD	Intercept	-0.006	-0.172	0.160	0.084	0.00	1.04	0.0	.945	.945	< . 001
FC5	PSD	Bladder Pain	-0.007	-0.018	0.005	0.006	1.44	1.04	1.4	.241	.796	.01
FC5	PSD	Menstrual Pain	0.004	-0.002	0.011	0.003	1.80	1.04	1.7	.189	.968	.012
FC5	PSD	Somatic Symptoms	-0.010	-0.083	0.062	0.037	0.08	1.04	0.1	.782	.931	.001
FC6	PSD	Intercept	0.008	-0.162	0.179	0.086	0.01	1.09	0.0	.924	.945	< . 001
FC6	PSD	Bladder Pain	0.010	-0.001	0.022	0.006	3.51	1.09	3.2	.075	.796	.022
FC6	PSD	Menstrual Pain	0.001	-0.006	0.008	0.003	0.15	1.09	0.1	.716	.968	.001
FC6	PSD	Somatic Symptoms	0.007	-0.068	0.082	0.038	0.04	1.09	0.0	.853	.931	< . 001
Fp1	PSD	Intercept	0.035	-0.150	0.221	0.094	0.18	1.29	0.1	.706	.779	.001
Fp1	PSD	Bladder Pain	0.006	-0.007	0.018	0.006	1.00	1.29	0.8	.381	.916	.005
Fp1	PSD	Menstrual Pain	-0.001	-0.008	0.007	0.004	0.03	1.29	0.0	.887	.968	< . 001
Fp1	PSD	Somatic Symptoms	0.054	-0.027	0.135	0.041	2.26	1.29	1.8	.188	.794	.012
Fp2	PSD	Intercept	0.092	-0.141	0.325	0.118	1.25	2.04	0.6	.435	.618	.004
Fp2	PSD	Bladder Pain	0.004	-0.011	0.020	0.008	0.59	2.04	0.3	.593	.974	.002
Fp2	PSD	Menstrual Pain	0.001	-0.009	0.010	0.005	0.05	2.04	0.0	.874	.968	< . 001
Fp2	PSD	Somatic Symptoms	-0.060	-0.161	0.042	0.052	2.74	2.04	1.3	.249	.794	.009
FT10	PSD	Intercept	0.152	-0.010	0.315	0.082	3.42	0.99	3.5	.065	.236	.024
FT10	PSD	Bladder Pain	-0.005	-0.016	0.006	0.006	0.83	0.99	0.8	.361	.916	.006
FT10	PSD	Menstrual Pain	0.001	-0.006	0.007	0.003	0.04	0.99	0.0	.833	.968	< . 001
FT10	PSD	Somatic Symptoms	-0.037	-0.108	0.034	0.036	1.07	0.99	1.1	.299	.794	.008
FT9	PSD	Intercept	0.067	-0.146	0.279	0.108	0.65	1.70	0.4	.536	.701	.003
FT9	PSD	Bladder Pain	0.003	-0.012	0.017	0.007	0.24	1.70	0.1	.706	.974	.001
FT9	PSD	Menstrual Pain	-0.002	-0.010	0.007	0.004	0.34	1.70	0.2	.657	.968	.001
FT9	PSD	Somatic Symptoms	0.080	-0.013	0.172	0.047	4.87	1.70	2.9	.093	.794	.02
Fz	PSD	Intercept	0.086	-0.128	0.300	0.108	1.08	1.72	0.6	.428	.618	.004

Fz	PSD	Bladder Pain	-0.005	-0.019	0.009	0.007	0.84	1.72	0.5	.486	.973	.003
Fz	PSD	Menstrual Pain	-0.001	-0.009	0.008	0.004	0.06	1.72	0.0	.852	.968	< . 001
Fz	PSD	Somatic Symptoms	-0.006	-0.099	0.088	0.047	0.03	1.72	0.0	.902	.931	< . 001
O1	PSD	Intercept	0.089	-0.045	0.222	0.067	1.15	0.67	1.7	.192	.383	.012
O1	PSD	Bladder Pain	0.007	-0.002	0.016	0.005	1.50	0.67	2.2	.137	.796	.015
O1	PSD	Menstrual Pain	-0.005	-0.010	0.001	0.003	2.05	0.67	3.1	.082	.968	.021
O1	PSD	Somatic Symptoms	0.006	-0.052	0.064	0.029	0.03	0.67	0.0	.838	.931	< . 001
O2	PSD	Intercept	0.075	-0.057	0.207	0.067	0.83	0.65	1.3	.262	.466	.009
O2	PSD	Bladder Pain	-0.006	-0.014	0.003	0.004	0.99	0.65	1.5	.22	.796	.01
O2	PSD	Menstrual Pain	0.001	-0.004	0.007	0.003	0.14	0.65	0.2	.643	.968	.002
O2	PSD	Somatic Symptoms	-0.006	-0.064	0.051	0.029	0.03	0.65	0.0	.827	.931	< . 001
Oz	PSD	Intercept	0.107	0.040	0.174	0.034	1.68	0.17	9.9	.002	.021	.065
Oz	PSD	Bladder Pain	0.005	0.001	0.010	0.002	0.83	0.17	4.9	.029	.796	.033
Oz	PSD	Menstrual Pain	-0.001	-0.004	0.002	0.001	0.12	0.17	0.7	.404	.968	.005
Oz	PSD	Somatic Symptoms	-0.005	-0.034	0.025	0.015	0.02	0.17	0.1	.75	.931	.001
P3	PSD	Intercept	0.100	-0.012	0.212	0.057	1.46	0.47	3.1	.081	.242	.021
P3	PSD	Bladder Pain	0.001	-0.007	0.008	0.004	0.02	0.47	0.1	.821	.974	< . 001
P3	PSD	Menstrual Pain	0.003	-0.001	0.008	0.002	0.85	0.47	1.8	.182	.968	.012
P3	PSD	Somatic Symptoms	-0.028	-0.077	0.021	0.025	0.61	0.47	1.3	.26	.794	.009
P4	PSD	Intercept	0.083	-0.025	0.192	0.055	1.01	0.44	2.3	.132	.283	.016
P4	PSD	Bladder Pain	-0.002	-0.009	0.005	0.004	0.11	0.44	0.3	.615	.974	.002
P4	PSD	Menstrual Pain	-0.003	-0.007	0.002	0.002	0.64	0.44	1.4	.231	.968	.01
P4	PSD	Somatic Symptoms	0.007	-0.041	0.054	0.024	0.03	0.44	0.1	.782	.931	.001
P7	PSD	Intercept	0.117	-0.001	0.235	0.060	2.02	0.52	3.9	.051	.235	.026
P7	PSD	Bladder Pain	0.003	-0.005	0.011	0.004	0.37	0.52	0.7	.401	.916	.005
P7	PSD	Menstrual Pain	-0.003	-0.008	0.002	0.002	0.83	0.52	1.6	.208	.968	.011
P7	PSD	Somatic Symptoms	0.026	-0.026	0.077	0.026	0.51	0.52	1.0	.323	.794	.007
P8	PSD	Intercept	0.199	0.085	0.313	0.058	5.84	0.49	11.9	.001	.012	.077
P8	PSD	Bladder Pain	0.000	-0.008	0.008	0.004	0.00	0.49	0.0	.974	.974	< . 001
P8	PSD	Menstrual Pain	0.001	-0.004	0.005	0.002	0.03	0.49	0.1	.814	.968	< . 001
P8	PSD	Somatic Symptoms	0.021	-0.029	0.071	0.025	0.34	0.49	0.7	.405	.868	.005

Pz	PSD	Intercept	0.156	0.053	0.260	0.052	3.60	0.40	9.0	.003	.021	.059
Pz	PSD	Bladder Pain	0.002	-0.005	0.009	0.004	0.17	0.40	0.4	.518	.974	.003
Pz	PSD	Menstrual Pain	0.003	-0.001	0.007	0.002	0.69	0.40	1.7	.192	.968	.012
Pz	PSD	Somatic Symptoms	-0.015	-0.060	0.030	0.023	0.16	0.40	0.4	.523	.93	.003
T7	PSD	Intercept	0.258	-0.018	0.534	0.140	9.79	2.86	3.4	.066	.236	.023
T7	PSD	Bladder Pain	-0.003	-0.022	0.015	0.009	0.38	2.86	0.1	.718	.974	.001
T7	PSD	Menstrual Pain	0.005	-0.006	0.016	0.006	2.08	2.86	0.7	.395	.968	.005
T7	PSD	Somatic Symptoms	-0.024	-0.145	0.096	0.061	0.45	2.86	0.2	.693	.931	.001
T8	PSD	Intercept	0.050	-0.120	0.220	0.086	0.37	1.08	0.3	.562	.701	.002
T8	PSD	Bladder Pain	-0.004	-0.016	0.007	0.006	0.61	1.08	0.6	.455	.97	.004
T8	PSD	Menstrual Pain	0.000	-0.007	0.007	0.003	0.00	1.08	0.0	.964	.968	< . 001
T8	PSD	Somatic Symptoms	-0.052	-0.126	0.022	0.038	2.08	1.08	1.9	.168	.794	.013
TP10	PSD	Intercept	0.098	-0.026	0.222	0.063	1.41	0.58	2.4	.122	.283	.017
TP10	PSD	Bladder Pain	0.000	-0.008	0.009	0.004	0.00	0.58	0.0	.934	.974	< . 001
TP10	PSD	Menstrual Pain	0.001	-0.004	0.006	0.003	0.14	0.58	0.2	.625	.968	.002
TP10	PSD	Somatic Symptoms	-0.004	-0.058	0.050	0.027	0.01	0.58	0.0	.881	.931	< . 001
TP9	PSD	Intercept	-0.072	-0.257	0.113	0.094	0.76	1.29	0.6	.444	.618	.004
TP9	PSD	Bladder Pain	0.000	-0.012	0.013	0.006	0.00	1.29	0.0	.956	.974	< . 001
TP9	PSD	Menstrual Pain	-0.003	-0.010	0.005	0.004	0.76	1.29	0.6	.443	.968	.004
TP9	PSD	Somatic Symptoms	0.007	-0.074	0.087	0.041	0.03	1.29	0.0	.874	.931	< . 001

Note. Results for the Intercept model are equivalent across all electrodes and are therefore not presented for each individual electrode; *df* for the numerator and denominator were 1 and 143, respectively; LL = lower level; UL = upper level.
